# Combining molecular and imaging metrics in cancer: radiogenomics

**DOI:** 10.1186/s13244-019-0795-6

**Published:** 2020-01-03

**Authors:** Roberto Lo Gullo, Isaac Daimiel, Elizabeth A. Morris, Katja Pinker

**Affiliations:** 1 Department of Radiology, Breast Imaging Service, Memorial Sloan Kettering Cancer Center, 300 E 66th St, New York, NY 10065 USA; 2 Department of Biomedical Imaging and Image-guided Therapy, Molecular and Gender Imaging Service, Medical University of Vienna, Waehringer Guertel 18-20, 1090 Wien, Austria

**Keywords:** Radiomics, Radiogenomics, Molecular profiling, Precision medicine

## Abstract

**Background:**

Radiogenomics is the extension of radiomics through the combination of genetic and radiomic data. Because genetic testing remains expensive, invasive, and time-consuming, and thus unavailable for all patients, radiogenomics may play an important role in providing accurate imaging surrogates which are correlated with genetic expression, thereby serving as a substitute for genetic testing.

**Main body:**

In this article, we define the meaning of radiogenomics and the difference between radiomics and radiogenomics. We provide an up-to-date review of the radiomics and radiogenomics literature in oncology, focusing on breast, brain, gynecological, liver, kidney, prostate and lung malignancies. We also discuss the current challenges to radiogenomics analysis.

**Conclusion:**

Radiomics and radiogenomics are promising to increase precision in diagnosis, assessment of prognosis, and prediction of treatment response, providing valuable information for patient care throughout the course of the disease, given that this information is easily obtainable with imaging. Larger prospective studies and standardization will be needed to define relevant imaging biomarkers before they can be implemented into the clinical workflow.

## Keypoints

- Current radiomic and radiogenomic studies are limited to few common cancers.
- Radiogenomics may provide accurate imaging biomarkers, substituting for genetic testing.
- Radiomics/radiogenomics biomarkers may predict risk and outcomes.
- Radiomics/radiogenomics biomarkers may be used to personalize treatment options.
- Larger prospective studies and standardization are needed to validate radiomics/radiogenomics biomarkers.

## Background

Personalized medicine is yielding increasingly precise disease treatments and prevention strategies for groups of individuals based on their genetic makeup, environment, and lifestyle. To enable personalized medicine, more precise and personalized genetic-based approaches (genomics, transcriptomics, proteomics, metabolomics, etc.) are used. In oncology, the goal of using such approaches is to allow more individual-level information rather than population-level or aspecific clinical information (tumor stage, age, gender, etc.) to select the most successful cancer treatment regimen for each patient [1].

Molecular tumor characterization can be performed using genomic and proteomic approaches, but this requires using tissue sampling from invasive surgery or biopsy [2]. However, even when molecular characterization is performed using tissue sampling, samples may not be accurate for the entire lesion as they are often obtained from a small portion of a heterogeneous lesion with inherent selection bias during biopsy [3]. Currently, large-scale genome cancer characterization that would allow genetic testing for every individual is not feasible due to high costs, the considerable time burden, and technically complex data analysis and interpretation [3].

Imaging can provide a more comprehensive view of the tumor in its entirety via radiomics and radiogenomics. Whereas radiomics analysis extracts large volumes of quantitative data from medical images and amalgamates these together with clinical and patient data into mineable shared databases [4–6], radiogenomics is the extension of radiomics through the combination of genetic and radiomic data. Detailed reviews of the process of radiomics analysis (image acquisition, volume of interest selection, segmentation, feature extraction and quantification, database building, classifier modeling, and data sharing) are described in detail by Pinker et al. [7], Gillies et al. [8], Sala et al. [5], and Lambin et al. [4].

Briefly, in radiomics, firstly, a region of interest (ROI) containing either the whole tumor or sub-regions within the tumor is identified from multimodality imaging data. ROIs are then segmented with operator edits and are eventually rendered in three dimensions (3D). High dimension features are extracted from these ROIs that include semantic and agnostic features [8]. Semantic features are morphological features that are commonly used in radiology reports to describe lesions such as size, location, vascularity, spiculation, and necrosis. Agnostic features are more complex mathematically extracted quantitative features which can be divided into first order statistical outputs (which describe distribution of value inside a single voxel), second order statistical outputs (describe interrelations between voxels), and higher order statistical outputs (extract repetitive and non-repetitive patterns within an image trough filter grids).

These features extracted from these rendered volumes generate a report, which is placed in a database along with other data, such as clinical and genomic data such as genes, mutations, and expression patterns.

Radiogenomics entails the correlation between quantitative or qualitative imaging features and the genomic data obtained from analysis of tissue as well as other clinical data, thus allowing the discovery of imaging surrogates that can serve as a substitute for genetic testing. Radiogenomics studies can be either exploratory or hypothesis-driven. Imaging features that are associated with single oncogenic defects of a tumor can be used to support treatment selection and monitoring as well as predict treatment outcomes [3, 5, 9, 10]. Hence, radiogenomics represents a promising novel approach to enable more personalized patient care [3, 4, 8, 9, 11–13].

## Main structure

Because genetic testing remains expensive, invasive, and time-consuming, and thus unavailable for all patients, radiogenomics may play an important role in providing accurate imaging surrogates which are correlated with genetic expression, thereby serving as a substitute for genetic testing [9, 13, 14]. These imaging surrogates can be used to predict response to therapy and the potential for early metastasis as well as to personalize treatment options [15–17].

So far, there are numerous genomic and clinical biomarkers identified from various adult cancers that have been collected in The Cancer Genome Atlas (TCGA). These biomarkers have been linked to the corresponding imaging data present in The Cancer Imaging Archive (TCIA) [17–19]. However, due to the lack of image sample registration (i.e., genetic test results cannot be matched to a specific location on imaging), the imaging data in the TCIA is so far limited for clinical use [3]. Given that imaging acquisition protocols are becoming more homogeneous and outcome data more robust, growing publicly available databases including the TCGA and TCIA will become more useful and will allow further radiogenomic studies [3].

Current radiomic and radiogenomic studies are limited to few types of common cancers. In this review, we will present the current data as pertains to radiomics and radiogenomics in glioblastoma multiforme (GBM), non-small cell lung cancer (NSCLC), hepatocellular carcinoma (HCC), intrahepatic cholangiocarcinoma, breast cancer (BC), prostate cancer, renal cell carcinoma, cervical cancer, and ovarian cancer and discuss their role and possible future applications in oncology.

### Brain

GBM remains the most common and the most fatal primary brain tumor in adults [20]. It is characterized by tremendous molecular and genomic heterogeneity, which leads to treatment resistance. Radiomics and radiogenomics research in the brain have thus far focused on GBM.

Through the TCGA, the genomic profile of GBM has been thoroughly assessed, resulting in its division into four distinct molecular subtypes: classical, mesenchymal, proneural, and neural. These subtypes are associated with different outcomes and tumor progression patterns [21, 22]. Another more recent stratification divided GBM into three core pathways according to RTK/RAS/PI [3] K, p53, and RB signaling alterations [23], showing a better correlation with outcomes. GBM magnetic resonance imaging (MRI) data from the TCIA has been matched with genetic data contained in the TCGA, enabling radiogenomics studies.

Using MRI, survival, and genetic data on 92 patients with GBM from the TCGA, Rao et al. [24] showed that a combination of three distinct features (volume-class, hemorrhage, and T1/fluid-attenuated inversion recovery (FLAIR)-envelope ratio) was able to stratify patient survival in a statistically significant manner. Between patients with poorer survival and patients with a better prognosis, there were significant differences in genes and microRNAs regulating invasion and proliferation (median difference of survival of 8 months).

Zinn et al. [25] used microRNA and genetic data of 78 GBMs from the TCGA to identify associations with quantitative MRI FLAIR features from the TCIA. The results suggested that MRI FLAIR can serve as an imaging surrogate for GBMs highly enriched in genes and microRNAs involved in cellular migration/invasion. In both discovery and validation sets, *POSTN* was the top upregulated gene and was associated with high FLAIR volumes, shorter progression-free survival, and shorter overall survival. *POSTN* upregulation is thought to induce tumor invasion through epithelial to mesenchymal transformation.

Colen et al. [26] reported in a study of 104 TCGA GBMs with MRI data from the TCIA that three MRI features could predict a worse prognosis: ependymal enhancement (10.6 versus 18.6 months, *p* = 0.0018), deep white matter tract involvement (10.9 months versus 19.9 months, *p* < 0.0008), and enhancement across midline (9 months versus 14.3 months (*p* < 0.0003). Ependymal enhancement and deep white matter tract involvement demonstrated significant association with mitochondrial dysfunction (*p* < 0.0001), *MYC* oncogene activation, and *NFKBIA* inhibition. A year later, using gene expression profiles from the TCGA and MRI data from the TCIA, Colen et al. [27] reported that patients with GBM with little necrotic component on MRI had a high prevalence of X-linked genes, while patients with high volumes of necrosis on MRI had a high prevalence of Y-linked genes. The authors also demonstrated that female patients with GBM characterized by low volumes of necrosis on MRI had a significant survival advantage.

To date, using TCGA/TCIA data or institutional data, tumor volume remains the most common MRI feature extracted from GBM and has been shown to be correlated with genomic data [25, 27–31]. Gutman et al. [31] carried out a volumetric analysis on the MRI of 76 GBMs profiled in the TCGA and found that tumors with *TP53* mutation had smaller enhancing and necrotic volumes (*p* ≤ 0.017) while tumors with *RB1* mutation were associated with less edema (*p* = 0.015). Using institutional data, Diehn et al. [32] found that the ratio of enhancing to non-enhancing volume was significantly correlated with *EGFR* overexpression (evaluated with immunohistochemistry) (*p* = 0.019). The enhancing phenotype was correlated with overexpression of angiogenesis and tumor hypoxia-related genes such as *VEGF*, *ADM*, and *PLAUR* (*p* = 0.012).

Barajas et al. [33] correlated MRI parameters with genetic features (investigated with RNA microarrays) in enhancing vs peritumoral non-enhancing GBM biopsy samples. Results showed that T2 dynamic susceptibility-weighted, perfusion-weighted, and diffusion-weighted measurements were different between biopsy regions and were correlated with histopathologic features of aggressiveness.

A study by Jamshidi et al. [34] in 23 patients with GBM identified associations between genes and MRI features such as contrast-to-necrosis ratio with *KLK3* and *RUNX3*, subventricular zone involvement with Ras oncogenes *RAP2* and *TYMS*, and vasogenic edema with oncogenes *FOXP1* and *PIK3IP1*.

Pope et al. [35] found that incomplete enhancing GBM on MRI was associated with increased levels of the oligodendroglioma markers *OLIG2* and *ASCL1* compared with completely enhancing imaging GBM. Histopathology confirmed this finding, showing a higher percentage of the oligodendroglioma histologic component in the incomplete enhancing group.

Pope et al. [36] also used magnetic resonance spectroscopy (MRS) in patients with glioma to measure the level of the oncometabolite 2-HG which is associated with mutations of *IDH1* and *IDH2*. Patients with *IDH1* mutation are more likely to progress to malignant gliomas. MRS detected elevated 2-HG levels in gliomas with *IDH1* mutations compared with those without the mutation (*p* = 0.003). Tumors with *IDH1* mutations showed elevated levels choline (*p* = 0.01) and decreased levels of glutathione (*p* = 0.03).

Hu et al. [37] demonstrated correlations between diffusion tensor imaging and dynamic susceptibility contrast perfusion metrics and mutations in *EGFR*, *PDGFRA*, *PTEN*, *CDKN2A*, *RB1*, and *TP53* (*p* < 0.03). Accuracies of the predictive models ranged from 37.5% for *TP53* to 87.5% for *RB1*. A similar study [38] involving MRI texture features was able to characterize local *EGFR* mutation status as well as predict patient survival in 65 GBMs.

In Jain et al. [39], CT perfusion parameters were correlated with angiogenesis-related genes. The authors found that 19 of 92 angiogenesis-related genes were significantly correlated with permeability surface area product and nine genes were significantly correlated with cerebral blood volume.

### Breast

Multiple studies have been conducted in the breast with promising results for BC analysis of genomic signatures, molecular subtype characterization, and clinically used recurrence scores. Most have relied mainly on tumor features in dynamic contrast-enhanced (DCE)-MRI, although diffusion-weighted imaging (DWI) has been used, for example, for characterization of molecular subtypes [40–42].

The first radiogenomic MRI study of the breast was published in 2012 by Yamamoto et al. [43] who investigated the potential of radiogenomics to correlate gene expression patterns with DCE-MRI characteristics. They found that 21 of 26 imaging characteristics were significantly associated with 71% of the approximately 52,000 variably expressed genes in BC. The same investigators also conducted another study [44] to examine the relationship between quantitative DCE-MRI imaging phenotypes, early metastasis, and long non-coding RNA expression using RNA sequencing. They found eight long non-coding RNAs that correlated with the enhancing rim fraction score which was in turn associated with early metastasis and poor metastasis-free survival in patients with BC.

Using a radiogenomics approach, Zhu et al. [45] investigated DCE-MRI characteristics (tumor size, shape, and morphology) of 91 BCs and their correlation with genomic features such as protein expression and mutations. Tumor size revealed that larger cancers have upregulated pathways, while blurred tumor margins and irregular shape were associated with more aggressive tumors.

Associations between MRI characteristics and molecular BC subtypes have been investigated in several studies. In a systematic review and meta-analysis published in 2014, Elias et al. [46] reported that higher enhancement within the tumor is associated with the luminal B subtype, while *HER2*-enriched cancers are more likely to show fast initial enhancement or wash-out kinetics with circumscribed margins. Elsewhere, triple-negative cancers have been associated with high T2 signal intensity and the presence of rim enhancement [47, 48]. In DWI, *HER2*-enriched tumors showed the highest ADC values, while luminal B/*HER2*-negative cancers showed the lowest [40–42].

Computer-extracted radiomic features have themselves been associated with molecular BC subtypes. Mazurowski et al. [49] showed that in DCE-MRI, extracted MRI features that relate to an increased ratio of tumor-to-background parenchymal enhancement were associated with *HER2*-positive cancers. This difference might be due to the increased vascularization found in *HER2*-positive subtypes mediated by *VEGF* which leads to increased vessel diameter, vascular permeability, and extracellular fluid. Grimm et al. [50] found correlations between extracted imaging features and luminal A and B breast cancer subtypes. In a more recent study by Grimm et al. [51], non-mass enhancement and qualitative BI-RADS descriptors were evaluated on DCE-MRI in 278 patients with breast cancer; results showed significant correlations between mass shape and basal cancers, mass margin and *HER2* cancers, and internal enhancement and luminal B cancers. In another study, Yamaguchi et al. [52] assessed the relationship between the delayed phase of enhancement of DCE-MRI and molecular subtypes, finding that *ER*-positive and/or *PgR*-positive and *HER2*-negative cancers demonstrated less washout. A recent study by Leithner et al. [53] showed that radiomic features extracted from DWI were able to separate breast cancers based on molecular subtype and receptor status with high accuracy (> 90%). Accuracy was superior for radiomics features extracted directly from the apparent diffusion coefficient (ADC) map.

**Fig. 1:**
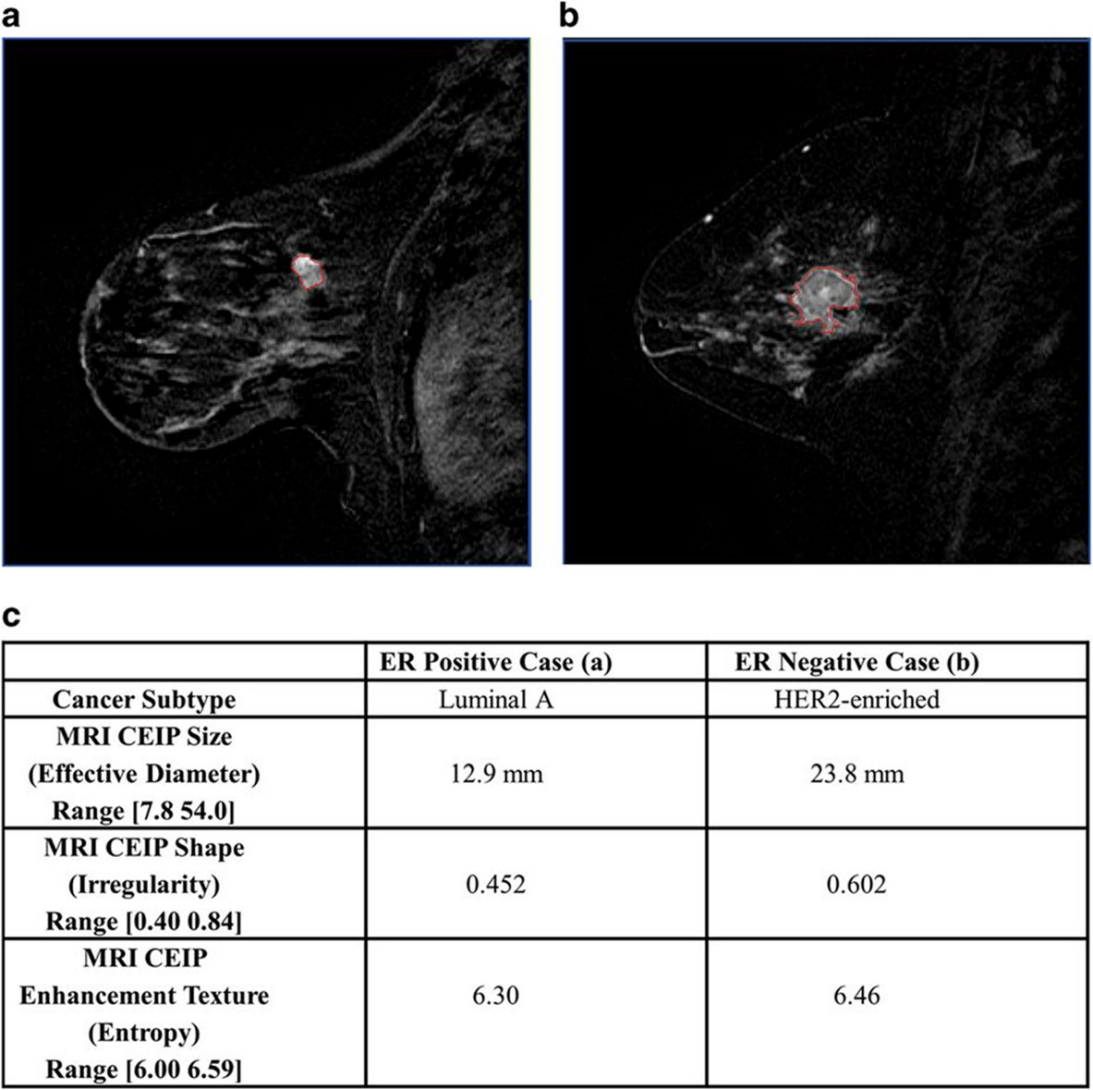
The computer segmentation method in example cases of one estrogen receptor positive tumor and one estrogen receptor negative tumor. The tumor segmentation outlines are shown along with computer-extracted image phenotype (CEIP) values (and ranges) for size, irregularity, and contrast enhancement heterogeneity. Reprinted under a Creative Commons license from: NPJ Breast Cancer. 2016;2. pii: 16012. Epub 2016 May 11. Quantitative MRI radiomics in the prediction of molecular classifications of breast cancer subtypes in the TCGA/TCIA data set. Li H, Zhu Y, Burnside ES, Huang E, Drukker K, Hoadley KA, Fan C, Conzen SD, Zuley M, Net JM, Sutton E, Whitman GJ, Morris E, Perou CM, Ji Y, Giger ML

Classifier models using tumor phenotypes to differentiate between molecular subtypes have been evaluated by Li et al. [54] with promising results (Fig. 1). However, in a similar study, Waugh et al. [55] found an accuracy of only 57.2%. More studies need to be conducted in this regard to validate these preliminary findings.

**Fig. 2:**
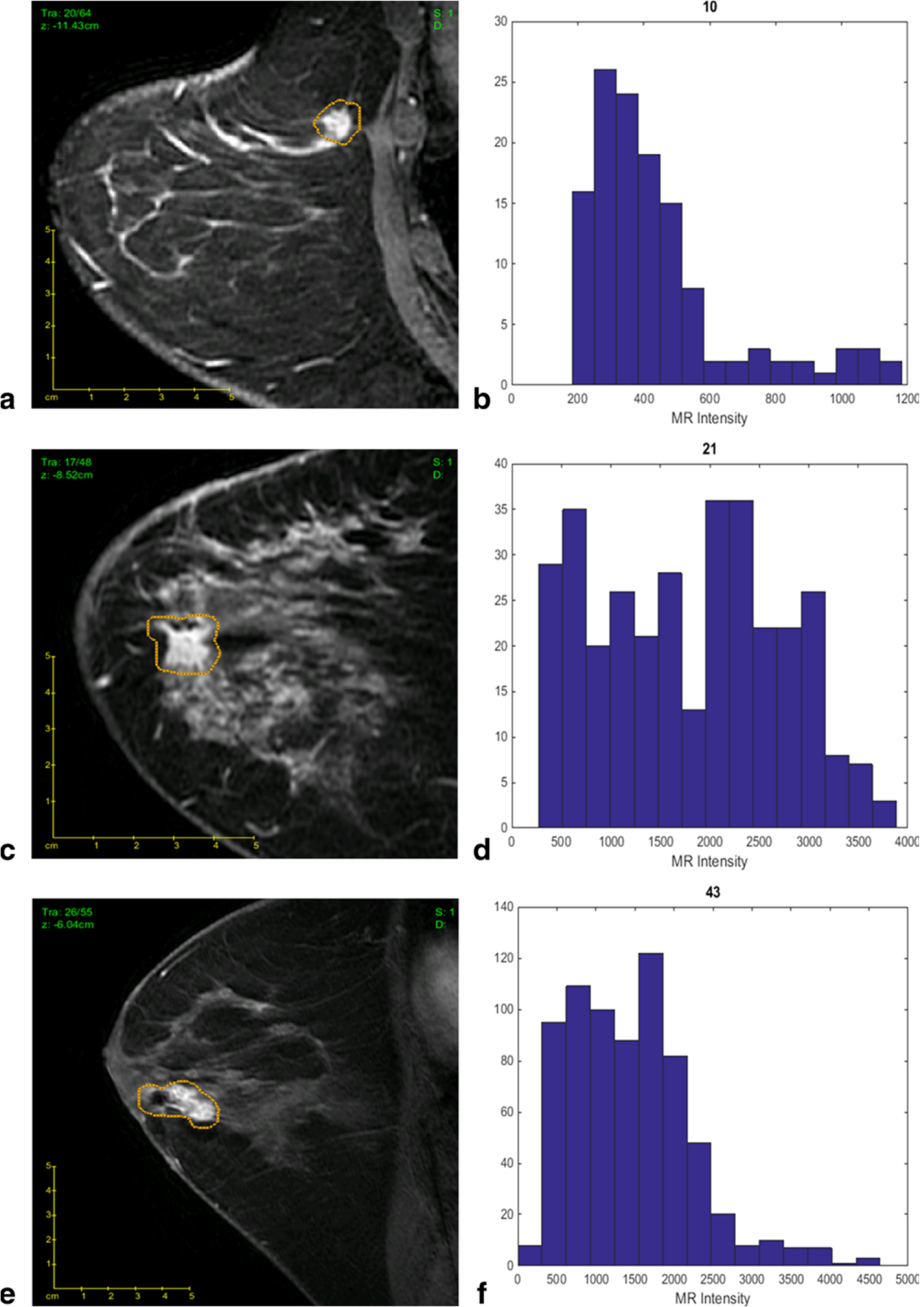
The best-fit linear regression model allows imaging features to differentiate tumors with different Oncotype Dx Recurrence Score (ODxRS). **a** Sagittal T1-weighted fat-suppressed post-contrast MRI of an invasive ductal nuclear grade 1 carcinoma with an ODxRS of 10 and (**b**) corresponding kurtosis histogram, which demonstrates the frequency of MR intensity. **c** Sagittal T1-weighted fat-suppressed postcontrast MRI of an invasive ductal nuclear grade 2 carcinoma with an ODxRS of 21 and (**d**) corresponding kurtosis histogram. **e** Sagittal T1-weighted fat-suppressed postcontrast MRI of an invasive ductal nuclear grade 3 carcinoma with an ODxRS of 43 and (**f**) corresponding kurtosis histogram. Reprinted with permission from: J Magn Reson Imaging. 2015 Nov;42 [5]:1398–406. doi: 10.1002/jmri.24890 Breast cancer subtype intertumor heterogeneity: MRI-based features predict results of a genomic assay. Sutton EJ, Oh JH, Dashevsky BZ, Veeraraghavan H, Apte AP, Thakur SB, Deasy JO, Morris EA.

**Fig. 3:**
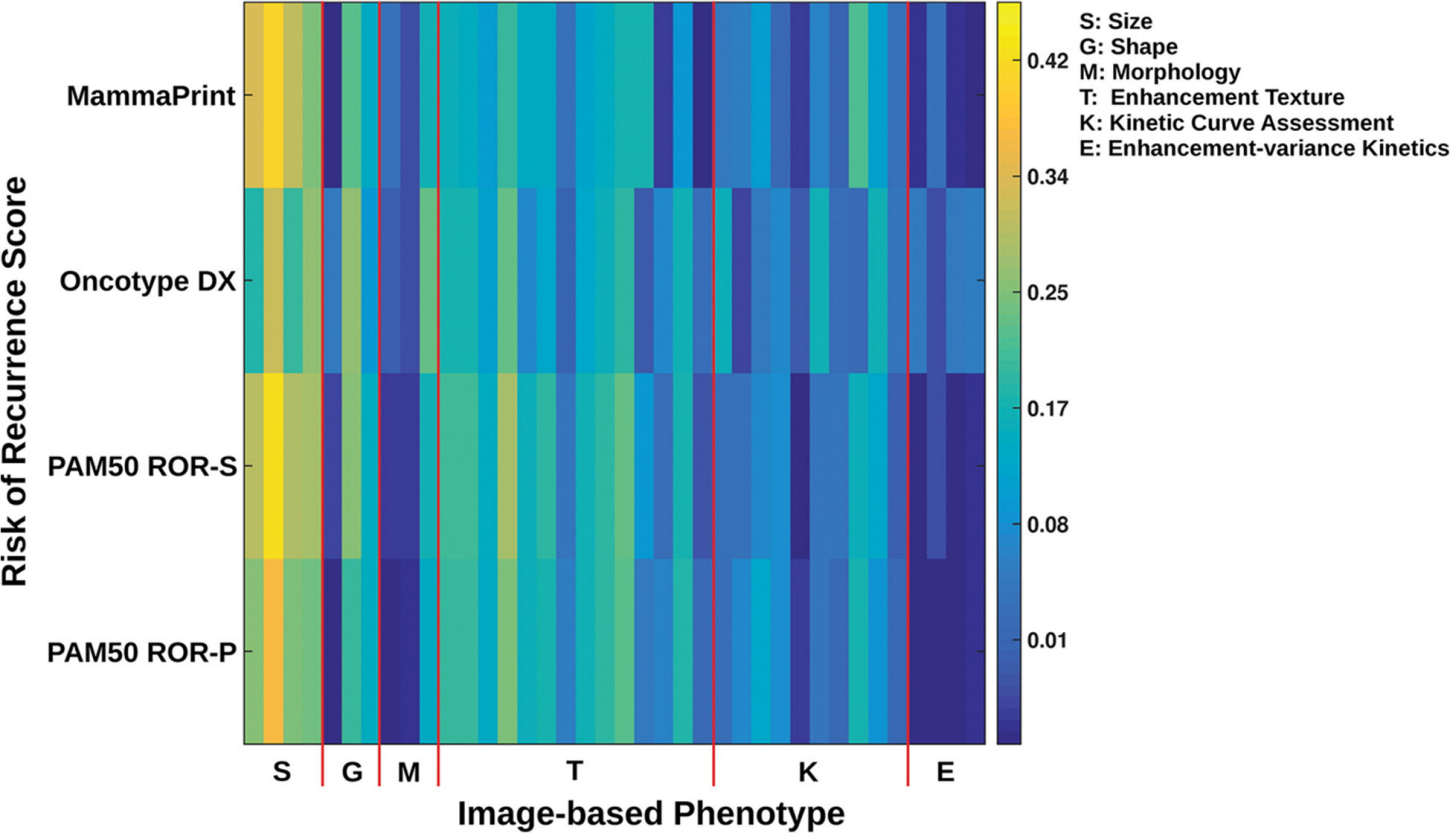
Correlation heat map based on univariate linear regression analysis between each individual MR imaging phenotype and the recurrence predictor models of MammoPrint, Oncotype DX, PAM50 ROR-S, and PAM50 ROR-P. In this color scale, yellow indicates higher correlation as compared with blue and the different gene assays served as the “reference standard” in this study. Some phenotypes correlate similarly (i.e., similar color on the color scale) across the risk estimate models, while others do not. Reprinted with permission from: Radiology. 2016 Nov;281 [2]:382–391. Epub 2016 May 5. MR Imaging Radiomics Signatures for Predicting the Risk of Breast Cancer Recurrence as Given by Research Versions of MammaPrint, Oncotype DX, and PAM50 Gene Assays. Li H, Zhu Y, Burnside ES, Drukker K, Hoadley KA, Fan C, Conzen SD, Whitman GJ, Sutton EJ, Net JM, Ganott M, Huang E, Morris EA, Perou CM, Ji Y, Giger ML

**Fig. 4:**
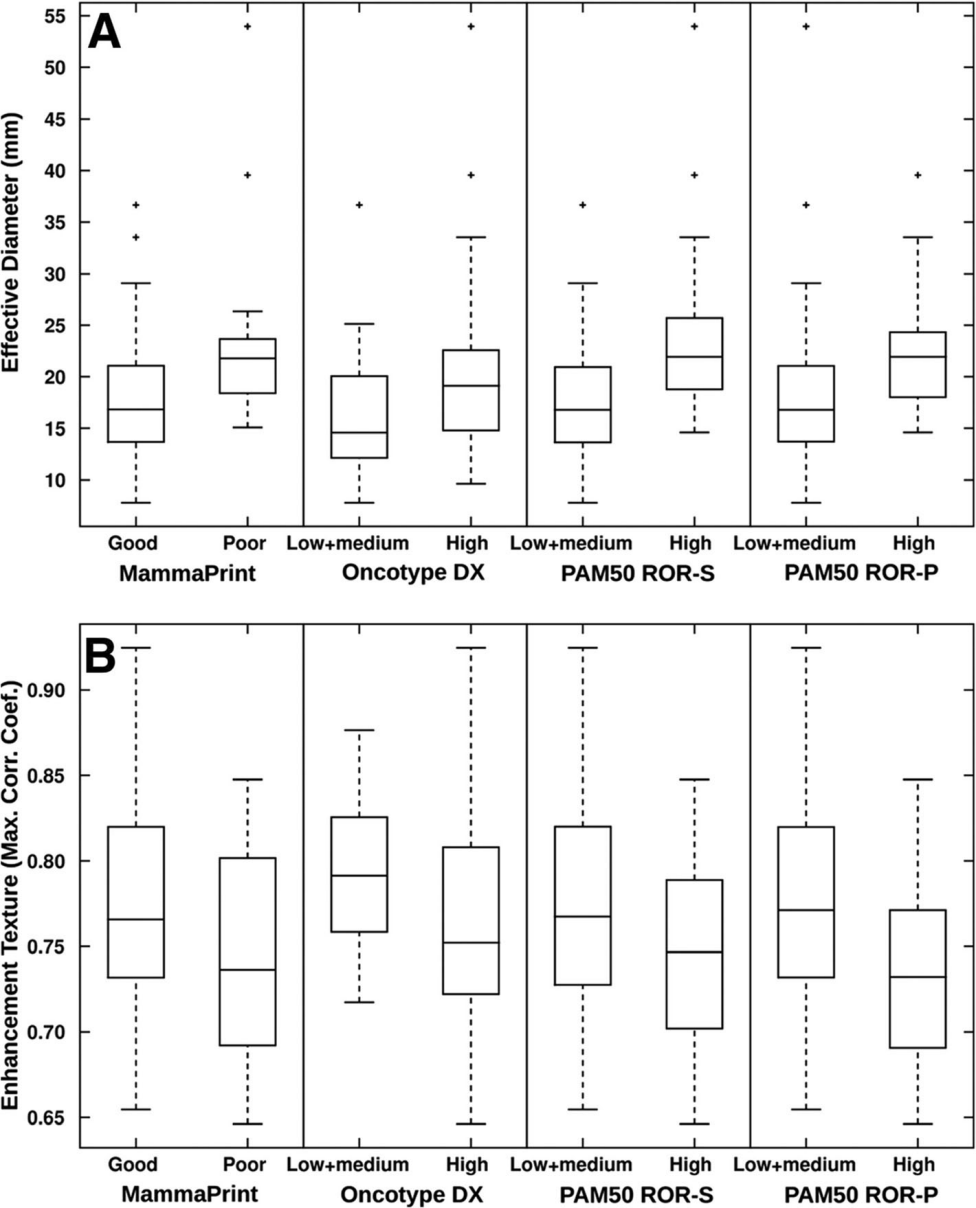
Box and whisker plots show the relationship of the MRI based phenotypes of (**a**) size (effective diameter) and (**b**) enhancement texture (maximum correlation coefficient) with the recurrence predictor models of MammaPrint, Oncotype DX, PAM50 ROR-S, and PAM50 ROR-P. A positive correlation between the selected MR imaging phenotypes of size (effective diameter) and negative correlation with enhancement texture (maximum correlation coefficient) and increasing levels of risk of recurrence for MammaPrint, Oncotype DX, PAM50 ROR-S, and PAM50 ROR-P were observed. A low value of this enhancement texture feature indicates a more heterogeneous enhancement pattern. Reprinted with permission from: Radiology. 2016 Nov;281 [2]:382–391. Epub 2016 May 5. MR Imaging Radiomics Signatures for Predicting the Risk of Breast Cancer Recurrence as Given by Research Versions of MammaPrint, Oncotype DX, and PAM50 Gene Assays. Li H, Zhu Y, Burnside ES, Drukker K, Hoadley KA, Fan C, Conzen SD, Whitman GJ, Sutton EJ, Net JM, Ganott M, Huang E, Morris EA, Perou CM, Ji Y, Giger ML

Previous studies have also correlated imaging characteristics with clinically available prognostic genomic assays which provide a clinical score for the risk of recurrence. As Oncotype Dx gene-expression score, MammaPrint, and PAM50 have been shown to predict recurrence in early-stage *ER*-positive/*HER2*-negative invasive cancers, these correlations between imaging characteristics and assay recurrence scores could have important implications in patient management. Woodard et al. [56] evaluated BC recurrence in *ER*-positive patients using OncotypeDx through the association of BI-RADS mammography and MRI features. Indistinct mass margins and fine linear branching calcifications on mammography were associated with a higher recurrence score, while breast density on mammography was inversely associated with the recurrence score. Spiculated mass margins and non-mass enhancement on MRI were associated with a lower recurrence score. Several radiomic imaging models assessing risk of recurrence using several assays have been developed [43–45, 57–65]. Combining imaging and pathology information, Sutton et al. [59] developed a model that correlated with the OncotypeDx Recurrence Score which was predictive of recurrence and therapeutic outcome (Fig. 2). A study by Li et al. [62] evaluated whether computer-extracted imaging phenotypes could predict cancer recurrence using MammaPrint, OncotypeDx, and PAM50/Prosigna. They found significant correlations between tumor size, heterogeneity, and higher risk for recurrence (Figs. 3 and 4). In this and other studies using radiomics computer-extraction methods [57, 62, 63], enhancement heterogeneity has been related to a high risk of recurrence in tumor assays. In two studies [57, 59], rapid contrast uptake predicted high-risk Oncotype Dx.

There is potential for radiogenomics studies regarding MR perfusion characteristics and genetic expression. One previous study [65] using genomics analysis has associated MR perfusion parameters with early metastasis or with differential gene expression when monitoring anti-*VEGF* treatment.

Yamamoto et al. [43] evaluated a qualitative imaging model including tumor heterogeneity and enhancement for prediction of expression of immune-response genes, and high-level analysis revealed 21 imaging traits that were globally significantly correlated with 71% of the total genes (3717/5231 genes) measured in BC patients.

### Gynecological tumors

To date, radiomics and radiogenomics have been applied in cervical cancer and in high grade serous ovarian cancer, providing valuable prognostic information.

DCE-MRI pharmacokinetic parameters have been associated with both genetic and outcome data in patients with cervical cancer. In Norway, Halle et al. [66] conducted an assessment of pre-treatment DCE-MRI pharmacokinetic parameters with global gene expression data in 78 patients who underwent chemotherapy. Through gene set analysis of 42/78 tumors, they found that the ABrix parameter correlated with hypoxia gene sets. Through immunohistochemistry analysis of the remaining 32/78 tumors, they found that a low ABrix was associated with upregulated *HIF1α* protein expression. A DCE-MRI hypoxia gene signature consisting of 31 hypoxia genes upregulated in tumors with low ABrix was constructed, showing prognostic value in an independent cohort of 109 patients. As reported by same group of authors [67] a year later, several pharmacokinetic parameters, also derived from pre-treatment DCE-MRI in 78 patients with cervical cancer who underwent chemotherapy, may be used to identify patients at risk of treatment failure: ABrix and Ktrans were associated with poor clinical outcome, while patients with high Kel had longer survival.

The TCGA Research Network previously introduced the Classification of Ovarian Cancer (CLOVAR) describing four prognostic genomic subtypes: differentiated, immunoreactive, mesenchymal, and proliferative [68, 69]. In a hypothesis-generating single-institution study, Vargas et al. [70] conducted a retrospective radiogenomics study of 46 patients with stage IIIC or IV high grade serous ovarian cancer. They reported that preoperative CT features evaluated by two radiologists were associated with the CLOVAR genomic subtypes of high grade serous ovarian cancer and were predictive of survival. Specifically, the presence of mesenteric infiltration and diffuse peritoneal involvement at baseline on CT were associated with CLOVAR mesenchymal subtype. The presence of mesenteric infiltration was also shown to provide important prognostic information as it correlated with shorter progression-free survival and overall survival. To validate the preliminary findings from this single-institution study, a multi-institutional study was then conducted by Vargas et al. [71] using TCIA CT images of 92 patients with high-grade serous ovarian cancer. The relationship between CT features and time-to-disease progression and CLOVAR profiles were assessed. The presence of peritoneal disease in the right upper quadrant, supradiaphragmatic lymphadenopathy, multiple peritoneal disease sites, and non-visualization of a discrete ovarian mass were associated with a shorter time-to-disease progression. The presence of multiple peritoneal disease sites and the presence of disease within the pouch of Douglas were associated with the mesenchymal subtype which has the worst prognosis among CLOVAR subtypes.

In another study by Vargas et al. [72], the authors used radiomics to derive inter-site spatial heterogeneity metrics across multiple metastatic lesions from preoperative CT of 38 patients with stage IIIC–IV high grade serous ovarian cancer. Several of these metrics were associated with the amplification of CCNE1, as well as a shorter overall survival and incomplete surgical resection. Previously, *CCNE1* amplification itself has been associated with higher chemoresistance [73] and treatment failure [74].

### Liver

HCC, the most common primary liver cancer, can be managed with a variety of treatment approaches such as embolization, radiation, surgery, and pharmacological therapy [7]. Currently, the approach is selected based on only the number and size of liver lesions [7]. In this context, radiomics and radiogenomics can serve an important role in helping to select the best clinical management for each patient [7]. The studies below indicate that they are particularly promising to allow for a better prediction of prognosis or treatment response.

The presence of microscopic venous invasion (MVI) is a well-established parameter associated with poor prognosis in HCC but its diagnosis is difficult with only conventional imaging [75, 76]. Pathologic examination of the explanted tissue after surgery is currently the only way to diagnose MVI. Since 2002, several groups have addressed this issue. Chen et al. [77] found a correlation between the presence of MVI and a 91-gene expression signature assessed through microarray analysis. Later, Segal et al. [78] identified two imaging features on CT—the presence of “internal arteries” and absence of “hypodense halos”— that were associated with these same 91 genes. In another study by Banerjee et al. [79] in 157 patients with HCC who eventually underwent surgical resection, these two imaging features along with “tumor–liver difference” were shown to be both highly predictive of the presence of histological MVI as well as associated with early disease recurrence and poor overall survival, thus showing that these features may be helpful to select candidates who will benefit less from surgical treatment or liver transplant. On MRI, rather than CT, Renzulli et al. [80] found that features including peritumoral enhancement and non-smooth margins were shown to be promising for the prediction of pathologic MVI in 125 patients with 140 nodules diagnosed with HCC.

Taouli et al. [81] found significant correlations between certain imaging traits in contrast-enhanced CT (26 patients) or MRI (12 patients) and gene signatures of aggressive HCC phenotype, with an infiltrative pattern on imaging having the highest number of positive associations. Although no correlations were found between gene expression signatures and enhancement ratios in this study, they proposed that their findings should be validated with DCE-MRI in the future to overcome the inherent limitations to measurements of tumoral enhancement on arterial and portal venous phases alone as used in their study.

When using multiparametric MRI, Hectors et al. [82] did not find differences among genetic subclasses in 14 patients, and none of the parameters could distinguish between HCC grades. However, they found significant correlations between several MRI parameters and individual gene expression levels, e.g., poor tumor perfusion on DCE-MRI correlated with high expression of *VEGF-A*. Multiparametric MRI with quantitative parameters is potentially a powerful tool for radiomics and radiogenomics study of HCC but the challenges to the reproducibility of advanced MRI techniques including DCE-MRI, DWI, and BOLD imaging in the liver presents a handicap.

Intrahepatic cholangiocarcinoma (ICC) is a less common tumor in the liver; therefore, only a few studies have been published to evaluate the relationship of ICC imaging features with genetic data. Sadot et al. [83] used texture analysis on the pre-treatment CT of 25 ICC patients, comparing the extracted quantitative and qualitative imaging phenotypes with hypoxia biomarkers such as *HIF-1α*, *VEGF*, *EGFR*, and CD24 measured in pre-treatment biopsies. Qualitative variables, including “tumor liver difference” and “attenuation heterogeneity,” were found to be correlated with *VEGF* expression, while CD24 expression was correlated with biliary dilatation.

**Fig. 5:**
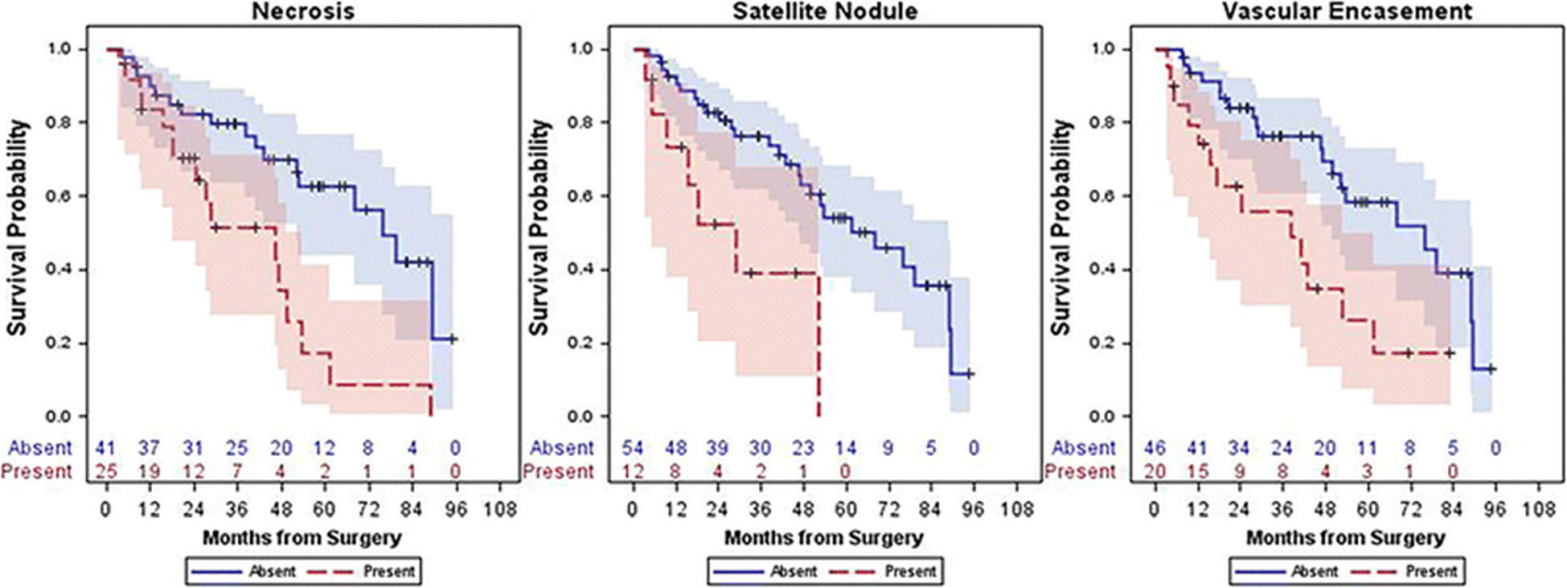
Kaplan–Meier survival curves which illustrate that the presence of necrosis, satellite nodules, and vascular encasement were all associated with decreased survival. Reprinted with permission from: Aherne EA, Pak LM, Goldman DA, Gonen M, Jarnagin WR, Simpson AL, and Do RK. Intrahepatic cholangiocarcinoma: can imaging phenotypes predict survival and tumor genetics? *Abdom Radiol,* 2018, 43 [10]:2665

**Fig. 6:**
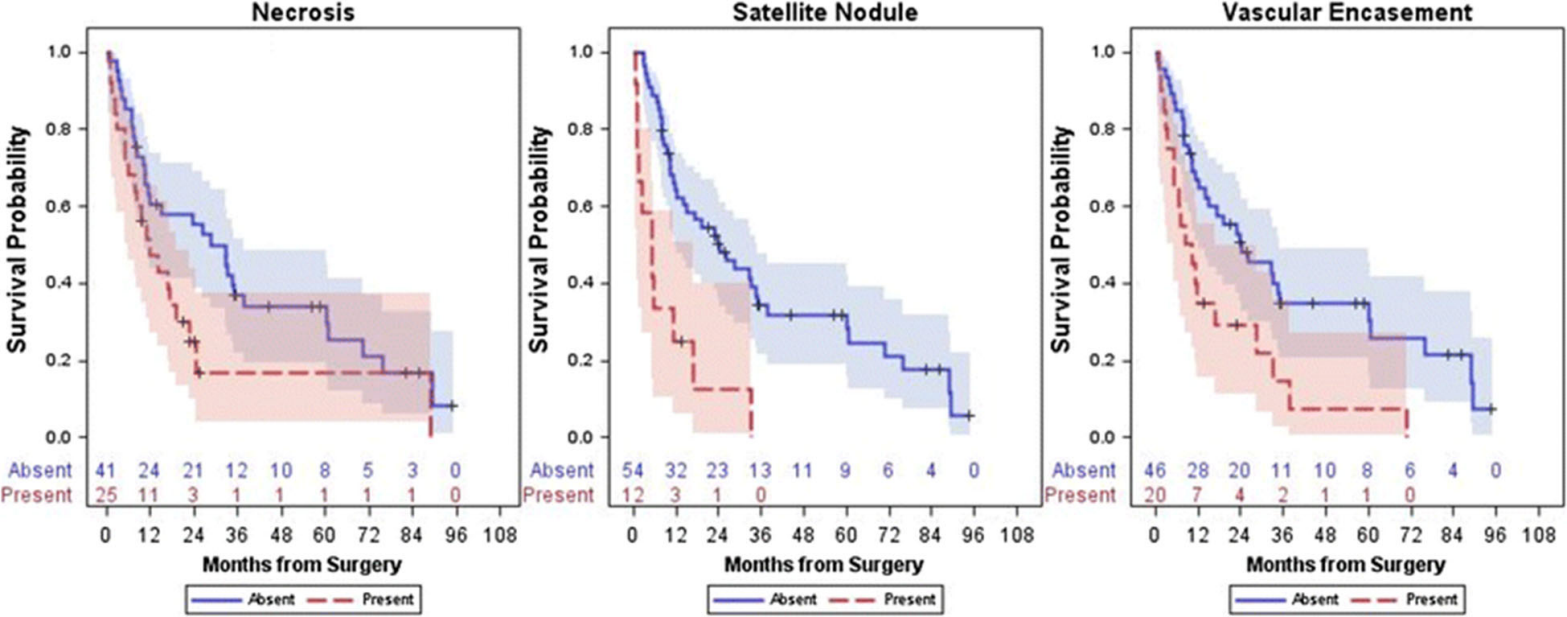
Kaplan–Meier survival curves which illustrate that the presence of satellite nodules and vascular encasement were associated with decreased disease-free survival. Reprinted with permission from: Aherne EA, Pak LM, Goldman DA, Gonen M, Jarnagin WR, Simpson AL, and Do RK. Intrahepatic cholangiocarcinoma: can imaging phenotypes predict survival and tumor genetics? *Abdom Radiol,* 2018, 43 [10]:2665

In 2011, Kim et al. [84] demonstrated that ICC enhancement in the arterial phase on CT was associated with fewer necrotic areas and longer disease-free survival after surgical resection. In 2017, Fujita et al. reached a similar in their study involving 47 ICC patients who underwent arterial phase CT. The 47 patients were divided into three groups: hypovascular, rim-enhancing, and hypervascular. Patients with hypovascular tumors had more instances of lymphatic, biliary, and perineural invasion and poorer disease-free survival than patients with hypervascular or rim-enhancing tumors [85]. More recently, Aherne et al. [86] identified a strong correlation between necrosis or vascular encasement on CT and decreased overall survival. A link was also identified between larger tumor sizes (or the presence of satellite nodules) and a reduction in progression-free survival (Figs. 5 and 6). This study also assessed associations between CT imaging features and genetic pathways (IDH1, chromatin, and RAS-MAPK) but did not find any significant associations.

### Kidney

The recent advances in genetics have led to the discovery of multiple mutations or genetic alterations in clear cell renal cell carcinomas (RCCs), including mutations or alterations of the genes encoding polybromo-1 protein (*PBRM1*), *BRCA1*-associated protein 1 (*BAP1*), SET domain containing 2 enzyme (*SETD2*), and lysine-specific demethylase 5C (*KDM5C*) [87–91], resulting in increased interest in the use of radiogenomics for assessing clear cell RCCs [92–95].

Even though the most common and well-known mutation identified in clear cell RCCs is the *VHL* tumor suppressor gene, it has no prognostic or predictive value in patients with clear cell RCC [96]. The second most commonly identified mutation in clear cell RCC is the *PBRM1* tumor suppressor gene [92, 97–99]. A recent meta-analysis [100] of 2942 RCC patients from seven studies reported that a mutation in or decreased expression of *PBRM1* is associated with poor survival, advanced TNM categories, advanced tumor stage, and a higher Fuhrman nuclear grade. A study by Kocak et al. [101] suggested that high-dimensional CT texture analysis is promising to distinguish clear cell RCCs with *PBRM1* mutation and those without *PBRM1* mutation.

Karlo et al. [93] conducted a preliminary radiogenomics study in 233 patients with clear cell RCC which showed associations between CT features and underlying mutations in several genes (*VHL*, *PBRM1*, *BAP1*, *SETD2*, and *KDM5C*). Well-defined tumor margins, nodular tumor enhancement, and intratumoral vascularization were associated with *VHL* mutations, while renal vein invasion was significantly associated with *KDM5C* and *BAP1* mutations. Mutations of *VHL* and *PBRM1* were more common in solid than in cystic tumors.

Using TCGA and TCIA data, Shinagare et al. [94] conducted a study of 103 clear cell RCCs (81 cases were evaluated with CT, 19 cases with MRI, and 3 cases with CT and MRI), showing that ill-defined tumor margins and calcifications were associated with *BAP1* mutation.

Several studies have shown that *KDM5C* mutation is associated with decreased survival in patients with clear cell RCC, though paradoxically it is associated with prolonged survival in patients with metastatic disease [102–105].

**Fig. 7:**
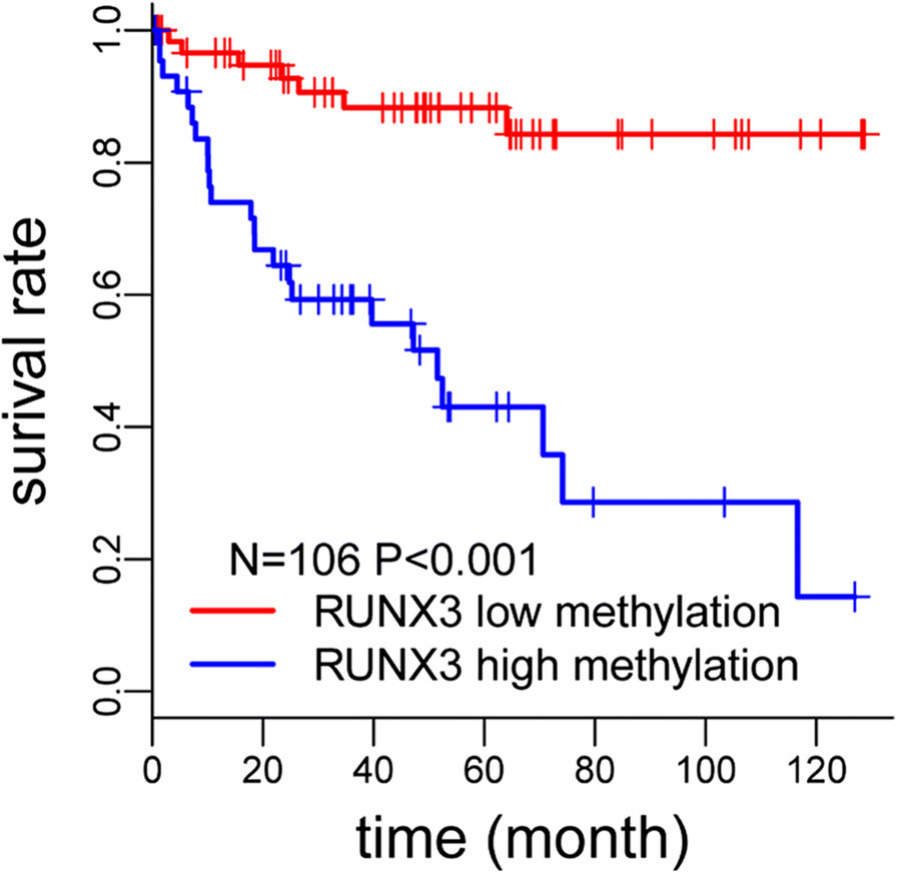
Univariate Cox regression analysis of prognostic factors for overall survival is summarized. Reprinted with permission from: Cen et al. [116] PubMed PMID: 30877466

**Fig. 8:**
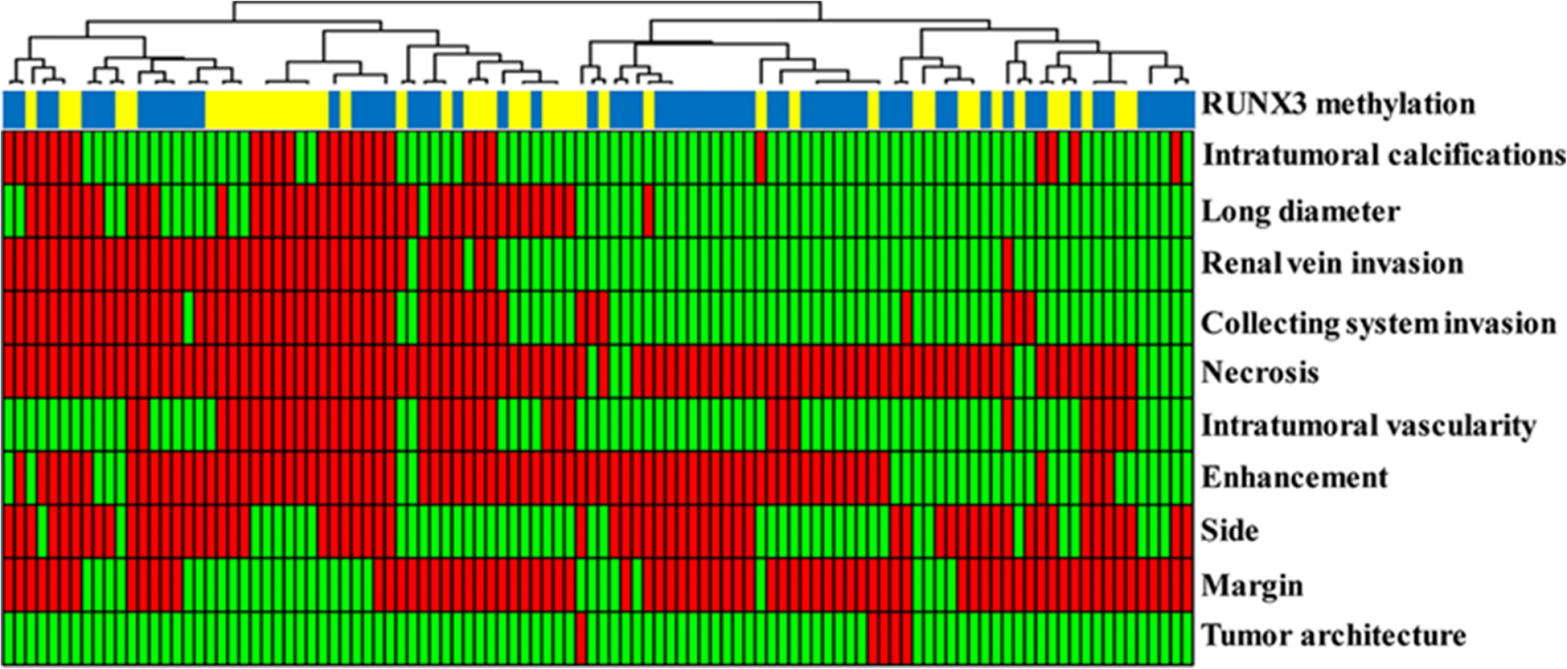
Hierarchical clustering yielded distinct groups of RUNX3 promoter methylation status and CT features. Red positive, green negative. Reprinted with permission from: Cen et al. [116] PubMed PMID: 30877466

Apart from associating imaging features with genetic mutations, they can also be associated with biomarkers such as DNA methylation, which is known to play a key role in cancer development and has potential prognostic and diagnostic value [106–109]. DNA methylation in tumor suppressor genes including *RUNX3* negatively impacts survival in some cancers [110–115]. An exploratory study by Cen et al. [116] divided RCC patients into high *RUNX3* methylation and low *RUNX3* methylation groups. Higher levels of *RUNX3* methylation were linked to decreased survival time (Fig. 7). Independent imaging predictors of high methylation of *RUNX3* such as ill-defined margins, high intratumoral vascularity, and left laterality of the lesions are shown in Fig. 8.

To build a radiogenomic risk score (RRS), a study by Jamshidi et al. [117] combined clinical data, genetic data extracted from genomic analysis, and preoperative CT data of clear cell RCC. Survival analysis confirmed that the high-RRS group had significantly lower disease-specific survival rates than the low RRS, independent of disease stage and grade.

### Lung

In the past decades, several subtypes of lung carcinoma harboring specific mutations (most notably *EGFR*, *KRAS*, and *ALK* mutations) have been identified, allowing for the development of therapies specifically targeting the mutated pathway [118].

The NSCLC lung cancer subtype is the leading cause of cancer death, accounting for more than 85% of all lung cancer cases. Genetic expression data of NSCLC is abundantly available in public databases. Approximately 15% of all NSCLCs in patients from European ethnicities and 50% of NSCLCs in never-smokers are *EGFR*-positive. The most frequent *EGFR* mutations (sensitizing activating mutations) are associated with tumor sensitivity to *EGFR* tyrosine kinase inhibitors (gefitinib, erlotinib, and afatinib) [119]. *ALK* mutations are less common (< 7% of all NSCLCs) and are more frequent in never/former smokers. Crizotinib was the first drug approved for NSCLC harboring *ALK* rearrangements, while ceritinib and alectinib have been approved only in the USA and Japan [118, 120, 121]. Approximately 20–25% of NSCLC harbor *KRAS* mutations and are associated with smoking and adenocarcinoma histology [122]. KRAS has been associated with poor response to standard treatments. Therapeutic strategies involving KRAS are currently under research [123].

Contradictory results have been found when considering the imaging features of different lung cancer subtypes. Glynn et al. did not find an association between CT imaging and *EGFR-* or *KRAS*-positive tumors [124]. Others, however, found associations. Lee et al. [125], using CT and 18-FDG PET/CT, demonstrated that tumors > 2.4 cm in diameter, an uptake of > 5.0, or ground glass opacity proportion of ≤ 50% within the lesion were associated with *EGFR* overexpression. The same group of authors [126] also showed that tumor morphology on CT could differentiate between the two most common subtypes of *EGFR* mutation, exon 19 deletions, and exon 21 mis-sense mutations; specifically, the ground glass portion of lesions were higher in lesions with exon 21 missense mutations compared with both wild type lesions and tumors with exon 19 deletions. Yano et al. [127] suggested an association between the size of a lesions ground glass component and the likelihood of an *EGFR* mutation [127].

In a study by Rizzo et al. [128], *EGFR* mutation was shown to be associated with CT features such as the presence of air bronchogram, pleural retraction, small lesion size, and absence of fibrosis, whereas *ALK* mutation was associated with pleural effusion. Round shape, nodules in non-tumor lobes, and smoking were variables linked to *KRAS* mutation. For this study, *ALK* gene rearrangement was detected by fluorescence in situ hybridization while *EGFR* and *KRAS* mutations were evaluated with DNA amplification of exons 18 through 21 and exon 2 and 3 respectively.

A study by Halpenny [129] showed that *ALK*-positive tumors tended to be bigger with a more solid consistency and involved more thoracic lymphadenopathy. This study showed no correlation between *ALK* mutation and presence of pleural effusion on CT.

Other, less frequent mutations such as *RET* and *ROS1*, which comprise 1–2% of all lung adenocarcinomas, have also been assessed for associations with imaging features. A study by Plodkowski et al. [130] included a group of patients with pathologically confirmed lung adenocarcinomas of any stage with a *RET* or *ROS1* rearrangement tested via fluorescence in-situ hybridization or next-generation sequencing and a control group of *EGFR*-positive lung cancers. CT features such as the presence of an effusion, lung metastases, adenopathy, and extra-thoracic disease were recorded. Peripheral tumors were seen most likely in patients with *ROS1* rearrangements (65% vs 32% in *EGFR*-positive cancers, *p* = 0.04). Solid tumors with spiculation were most likely *RET*- and *ROS1*-rearranged and *EGFR*-mutant, and these rarely presented with cavitations or calcifications.

Nair et al. [131] have examined differential genome wide expression across varying FDG uptake levels in patients with NSCLC to identify individual genes and gene expression signatures associated with prognostically relevant FDG uptake features. Their analysis suggested an existing correlation between NFΚB signaling and FDG uptake. NFΚB signaling seems to be increased in inflammatory and malignant conditions since it is enhanced by lactate as a result of glycolysis [132].

### Prostate

Prostate cancer is the most common cancer in men in the western world [133, 134]. However, only some prostate cancers exhibit a particularly more aggressive behavior, where they tend to metastasize and resist treatment [135]. Early detection of the more aggressive prostate cancers is important to optimally manage this subgroup [7]. To date, prostate risk stratification relies on clinical examination, biopsy data (e.g., Gleason grade), and serological markers (e.g., prostate-specific antigen) [136]. Genomic [137] and quantitative MRI-derived imaging biomarkers [138] have been shown to be helpful, not only in risk stratification, but also in detection [139, 140], staging [141], characterization [142–145], and treatment planning and follow-up [146, 147], thus setting the stage for radiogenomic studies.

**Fig. 9:**
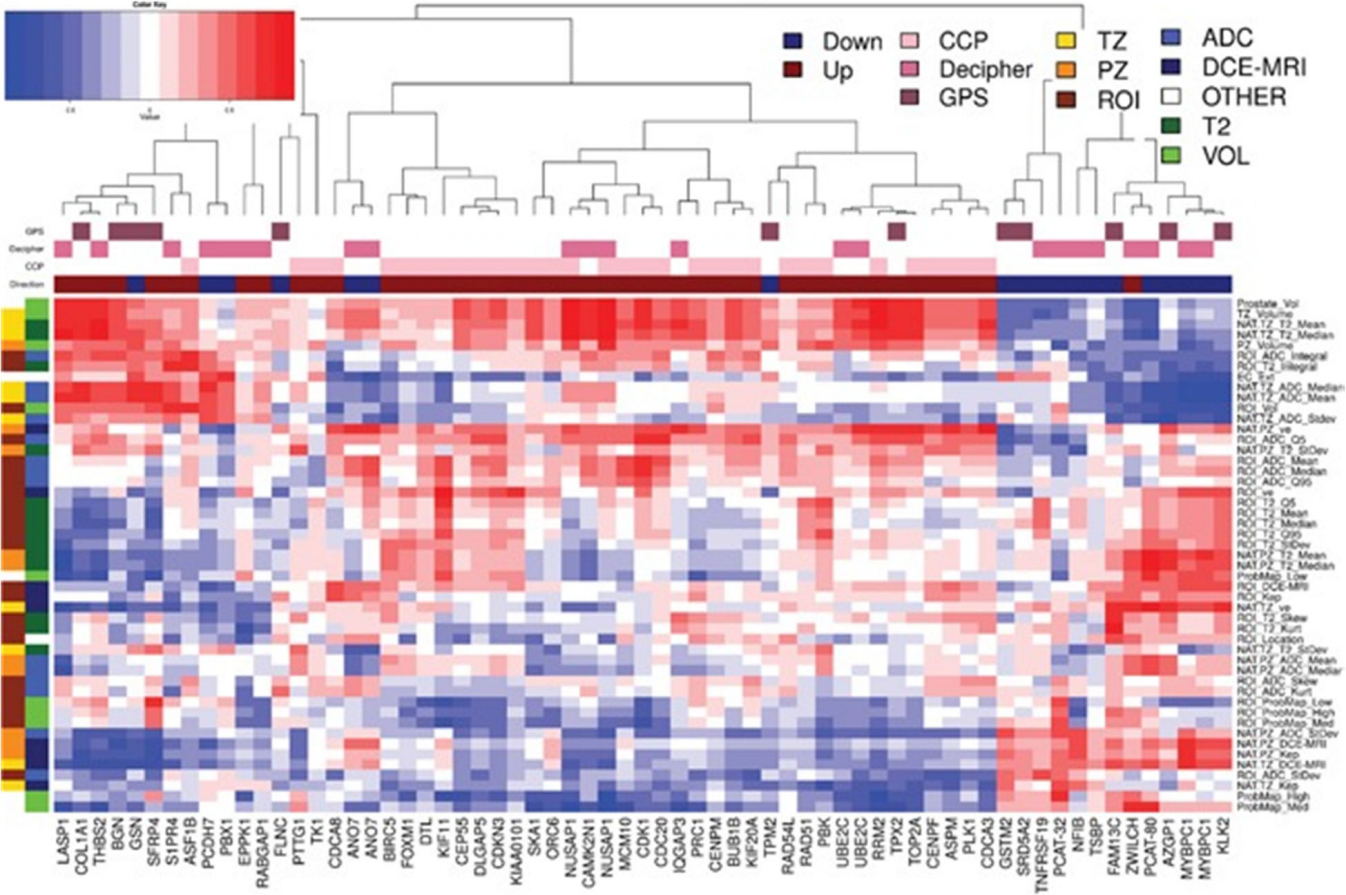
Pearson’s correlation analysis of imaging features and 65 genes from commercially available prostate cancer classifiers. Hierarchical clustering on Pearson’s correlation distance between radiomic features and genes from commercially available prostate cancer classifiers: CCP (Cell Cycle Progression), Decipher and GPS (Genomic Prostate Score). Genes in these signatures that are up-expressed in aggressive cancers are indicated by a dark red box over the gene’s column while those that are down-expressed are indicated with a blue box. Groups of radiomic features are indicated along the dendrogram on the left. Group1 (left) connects the radiomic feature with location (TZ, PZ and ROI); group 2 is related to the image modality/function: T2w, ADC, and DCE-MRI. Reprinted under a creative commons license from: Buerki C, Castillo R, Jorda M, Ashab HA, Kryvenko ON, Punnen S, Parekh D, Abramowitz MC, Gillies RJ, Davicioni E, Erho N, Ishkanian A. Association of multiparametric MRI quantitative imaging features with prostate cancer gene expression in MRI-targeted prostatebiopsies. Oncotarget. 2016 Aug 16;7 [33]:53362–53,376. doi: 10.18632/oncotarget.10523

Stoyanova et al. [15] hypothesized that radiomic features may be used to characterize imaging phenotypes or habitats in the prostate, thereby improving risk stratification. Distinct habitats on multiparametric MRI analysis were identified based on reduced diffusion and increased perfusion. From radiogenomic analysis, they reported that several radiomic imaging features were significantly associated with genes related to aggressive behavior (Fig. 9), and ADC values in particular were the most strongly associated with distinct biological processes.

Renard-Penna et al. [16] correlated MRI features (Prostate Imaging Reporting and Data System (PI-RADS) scores, lesion diameter, and mean ADC values) in 106 suspicious lesions with the Prolaris® Cell Cycle Progression score, finding a significant correlation between PI-RADS and CCP scores (*ρ* = 0.26, *p* = 0.007).

McCann et al. [148] correlated MRI features from 45 peripheral zone prostate cancer lesions in 30 patients with *PTEN* expression on prostatectomy specimens. They found only a weak correlation between *k*_*ep*_ and *PTEN* expression (*r* = − 0.35, *p* = 0.02).

Jamshidi et al. [149] conducted a study whereby spatial mapping between pre-operative multiparametric MRI and the resected prostate gland was performed. Whole-exome DNA sequencing data was performed on multiple regions of interest representing abnormal vs. abnormal regions. MRI features identified high-grade lesions correctly in all patients. However, there were no significant differences in mutation profiles between histopathologically normal tissue, high-grade prostate cancer, MRI-normal, and MRI-suspicious regions (*p* = 0.3). The authors suggested that the baseline mutation spectrum among non-cancerous tissue within prostate may be wider than hypothesized.

## Challenges

Radiogenomics is an emerging field that correlates tumor genotypes with imaging phenotypes. Over the past 10 years, numerous studies have been published on radiogenomics of various cancers, yet the implementation of radiogenomic in clinical practice is still not routinely done. This is due to several limitations associated with radiogenomic analysis.

Gene expression and signaling pathways are extremely complex, and it is difficult to match the large amount of data from whole-genome sequencing with imaging data (in literature there are only few studies that use the whole genome data). The dimensionality of genomic data should be reduced to match that obtained from imaging studies.

Differences in quantitative imaging features are not only related to gene expression but can also be related to other factors such as patient characteristics or imaging technique. Inter- and intra-institutional heterogeneity of datasets due to different hardware and scan protocols limits the generalizability of results. The limitations associated with interobserver variation make qualitative imaging features even less preferable.

Another factor limiting generalizability and reproducibility of the results stems from the often-small patient cohort and the retrospective nature of radiogenomic studies.

Larger prospective studies and standardization will be necessary to validate the potential of radiogenomics, define relevant imaging biomarkers, and define which radiogenomics associations can be meaningfully implemented in the clinical routine.

## Conclusion

Radiomics and radiogenomics are promising to increase precision in diagnosis, assessment of prognosis, and prediction of treatment response, providing valuable information for patient care throughout the course of the disease, given that this information is easily obtainable with imaging. With personal medicine playing an increasing role in clinical practice, radiogenomics in particular can allow fast and non-invasive genotype identification and be applied to all cancer types. Further research in radiogenomics should be conducted to obtain larger datasets with more accurate information for the standardization to provide meaningful and clinically applicable results, and standardization will be necessary to validate the potential of radiogenomics and to define relevant imaging biomarkers before they can be implemented into the clinical workflow.

## Data Availability

Not applicable

## Abbreviations

ADC: Apparent diffusion coefficient
BC: Breast cancer
CCP: Cell Cycle Progression
CLOVAR: Classification of Ovarian Cancer
CT: Computed tomography
DCE: Dynamic contrast-enhanced
DSC: Dynamic susceptibility contrast
DTI: Diffusion tensor imaging
DWI: Diffusion-weighted imaging
FLAIR: Fluid-attenuated inversion recovery
GBM: Glioblastoma multiforme
HCC: Hepatocellular carcinoma
ICC: Intrahepatic cholangiocarcinoma
MRI: Magnetic resonance imaging
MVI: Microvascular invasion
PI-RADS: Prostate Imaging Reporting and Data System
RCC: Renal cell carcinoma
ROI: Region of interest
RRS: Radiogenomic risk score
TCGA: The Cancer Genome Atlas
TCIA: The Cancer Imaging Archive
TTP: Time-to-disease progression
